# Cardioselective peripheral noradrenergic deficiency in Lewy body synucleinopathies

**DOI:** 10.1002/acn3.51243

**Published:** 2020-11-20

**Authors:** Guillaume Lamotte, Courtney Holmes, Patricia Sullivan, Abhishek Lenka, David S. Goldstein

**Affiliations:** ^1^ Clinical Neurosciences Program (CNP) Division of Intramural Research (DIR) National Institute of Neurological Disorders and Stroke (NINDS) National Institutes of Health (NIH) Bethesda Maryland USA; ^2^ Autonomic Medicine Section CNP/DIR/NINDS/NIH Bethesda Maryland USA; ^3^ Department of Neurology Medstar Georgetown University Hospital Washington District of Columbia USA

## Abstract

**Objective:**

Lewy body (LB) synucleinopathies such as Parkinson’s disease (PD) entail profound cardiac norepinephrine deficiency. The status of sympathetic noradrenergic innervation at other extracranial sites has been unclear. Although in vivo neuroimaging studies have indicated a cardioselective noradrenergic lesion, no previous study has surveyed peripheral organs for norepinephrine contents in LB diseases. We reviewed ^18^F‐dopamine (^18^F‐DA) positron emission tomographic images and postmortem neurochemical data across several body organs of controls and patients with the LB synucleinopathies PD and pure autonomic failure (PAF) and the non‐LB synucleinopathy multiple system atrophy (MSA).

**Methods:**

^18^F‐DA–derived radioactivity in the heart, liver, spleen, pancreas, stomach, kidneys, thyroid, and submandibular glands were analyzed from 145 patients with LB synucleinopathies (112 PD, 33 PAF), 74 controls, and 85 MSA patients. In largely separate cohorts, postmortem tissue norepinephrine data were reviewed for heart, liver, spleen, pancreas, kidney, thyroid, submandibular gland, and sympathetic ganglion tissue from 38 PD, 2 PAF, and 5 MSA patients and 35 controls.

**Results:**

Interventricular septal ^18^F‐DA–derived radioactivity was decreased in the LB synucleinopathy group compared to the control and MSA groups (P < 0.0001 each). The LB and non‐LB groups did not differ in liver, spleen, pancreas, stomach, or kidney ^18^F‐DA–derived radioactivity. The LB synucleinopathy group had markedly decreased apical myocardial norepinephrine, but normal tissue norepinephrine in other organs. The MSA group had normal tissue norepinephrine in all examined organs.

**Interpretation:**

By in vivo sympathetic neuroimaging and postmortem neurochemistry peripheral noradrenergic deficiency in LB synucleinopathies is cardioselective. MSA does not involve peripheral noradrenergic deficiency.

## Introduction

Lewy body (LB) diseases such as sporadic Parkinson’s disease (PD) are characterized by cytoplasmic intraneuronal deposition of the protein alpha‐synuclein in the brain and autonomic nervous system and are considered to be in a family of disorders termed synucleinopathies.^1^ LB synucleinopathies feature severe myocardial norepinephrine depletion,^2^ implying that the disease process is not confined to the central nervous system and involves pathology in postganglionic sympathetic noradrenergic nerves. In contrast, the non‐LB synucleinopathy multiple system atrophy (MSA) is characterized by alpha‐synuclein deposits in glial cells in the central nervous system^3^ and typically does not involve peripheral noradrenergic deficiency.^4^

Studying peripheral noradrenergic innervation in LB forms of synucleinopathy is important scientifically and clinically because autonomic involvement seems to occur early in the pathogenetic sequence leading to relatively morbid “body‐first” PD.^5^, ^6^, ^7^, ^8^ Several studies have reported alpha‐synuclein aggregates in the enteric nervous system, which might then be transmitted in a prion‐like manner to the CNS.^9^, ^10^, ^11^

Pathological studies in PD have yielded inconsistent findings about which plexuses and neurons in the periphery are most affected.^12^ Whether patients with LB synucleinopathies have decreased norepinephrine contents in organs other than the heart has been unknown. Neuroimaging studies have provided evidence for decreased sympathetic innervation in the thyroid gland, submandibular gland, or renal cortex of patients with PD; however, postmortem data have so far not been reported.^13^, ^14^ Of particular interest is the pancreas, where autonomic neuronal, adrenomedullary hormonal, and autocrine–paracrine catecholaminergic systems converge,^15^, ^16^, ^17^ yet to date no study has described neuroimaging or postmortem neurochemical data about the pancreas in synucleinopathies.

The purpose of this study therefore was to assess in vivo and postmortem cardiac and extracardiac sympathetic noradrenergic innervation in individuals with synucleinopathies and controls. ^18^F‐dopamine positron emission tomography (^18^F‐DA PET) scanning was used to visualize peripheral noradrenergic innervation in organs such as the heart, liver, spleen, stomach, kidneys, thyroid, submandibular glands, and pancreas. In largely separate cohorts, postmortem tissue NE contents in the heart, liver, spleen, kidneys, thyroid gland, submandibular glands, pancreas, and sympathetic ganglia were assayed.

We hypothesized that the results of in vivo neuroimaging and postmortem neurochemistry would agree in terms of the extent of peripheral noradrenergic deficiency in LB synucleinopathies. Based on the in vivo neuroimaging literature, we expected that LB synucleinopathy patients would have severely decreased norepinephrine content in the apical myocardium and less decreased or normal norepinephrine contents in other organs. We also expected that in most MSA patients there would be normal norepinephrine contents in all extracranial organs including the heart.

## Methods

### Subjects

All the living subjects in this study gave written informed consent to participate in protocols approved by the Institutional Review Board of the National Institute of Neurological Disorders and Stroke (NINDS). We reviewed the neuroimaging data from all ^18^F‐DA PET scans done under protocols of the Autonomic Medicine Section (formerly Clinical Neurocardiology Section) at the National Institutes of Health (NIH) between 1990 and 2020. Patients had been referred for evaluation of chronic autonomic failure or were healthy volunteers or subjects with chronic hypertension who were included in a protocol at the NIH that included ^18^F‐DA PET scanning.

When a subject underwent more than one ^18^F‐DA PET scan, we averaged the radioactivity concentrations over time for the same organ of interest. Autonomic function testing to identify neurogenic orthostatic hypotension included continuous blood pressure recording associated with the performance of the Valsalva maneuver and orthostatic plasma catechols. A diagnosis was assigned to each patient based on the medical and neurological history and the physical and neurological examinations, supported by the results of specialized tests described below.

### Lewy body synucleinopathies

Patients with PD and pure autonomic failure (PAF) were included in the LB synucleinopathy group. The study did not include a patient cohort with LB dementia.

#### Parkinson’s disease

A diagnosis of PD was assigned based on at least three of the following four clinical criteria: bradykinesia, resting tremor, cogwheel rigidity, and good response of the movement disorders to levodopa treatment.^18^ Supportive clinical laboratory findings included central dopaminergic deficiency as indicated by low putamen/occipital cortex ratios of ^18^F DOPA‐derived radioactivity.^19^ The PD cohort was stratified into two groups: those with neurogenic orthostatic hypotension (PD + OH, N = 52) and those without neurogenic orthostatic hypotension (PD No OH, N = 60).

#### Pure autonomic failure

A diagnosis of PAF (N = 33) was assigned based on chronic, persistent neurogenic OH,^20^ without a known cause (e.g., diabetic autonomic neuropathy, autoimmune autonomic ganglionopathy) and supported by clinical laboratory evidence of sympathetic noradrenergic deficiency.^4^, ^21^

### Multiple system atrophy

The clinical diagnosis of probable MSA was made based on consensus criteria.^22^ The MSA cohort was divided into two groups, that is, parkinsonian (MSA‐P, N = 68) and cerebellar (MSA‐C, N = 17).

### Controls

A control group consisted of 67 healthy volunteers and seven subjects with chronic hypertension who were referred for evaluation prior to treatment with sympathectomy.

### ^18^F‐Dopamine positron emission tomographic scanning

PET scans were acquired on a GE Advance Tomograph (GE Healthcare) prior to January 2016 and on a Siemens PET/CT scanner after the GE Advance scanner was retired in January 2016. ^18^F‐DA PET scanning was done as described previously.^23^ Briefly, ^18^F‐DA–derived radioactivity was recorded for the 5‐minute frame with a midpoint about 8 minutes after initiation of the 3‐minute infusion of 1 mCi of the tracer. Radioactivity concentrations in nCi/cc in regions of interest were adjusted for the radioactivity dose in mCi per kg body mass and expressed in units of nCi‐kg/cc‐mCi.

PET images were analyzed with Pixelwise modeling computer software (PMOD 2.61; PMOD Group). The organs of interest were visualized easily on ^18^F‐DA PET scans, and when the images were acquired on a PET/CT scanner the PMOD image registration/fusion tool was used to confirm the localization of each organ of interest on CT. The organs of interest were the cardiac interventricular septum, left ventricular (LV) chamber, liver, spleen, pancreas, stomach, renal cortex, renal pelvis, thyroid, and submandibular glands. We placed regions of interest within the borders of the organs. In dynamic emission scans of the thoraco‐abdominal region, regions of interest were drawn on transverse tomographic slices of the interventricular septum, LV chamber, liver, spleen, renal cortex, renal pelvis, and pancreas and coronal tomographic slices of the stomach. For the stomach, the inner region of interest was the lumen and the outer region of interest was the margin of the stomach. We subtracted the inner region of interest from the outer region of interest in order to assess radioactivity concentration in the stomach wall. For the renal cortex and renal pelvis, we exploited the fact that ^18^F‐DA–derived radioactivity always appears in the renal cortex before it appears in the renal pelvis. In static emission scans of the head and neck, circular regions of interest were drawn on transverse tomographic slices of the submandibular glands and thyroid.

The mean ± SEM concentrations of ^18^F‐DA–derived radioactivity in the various organs were compared among the subject groups. Differences between groups were assessed by Dunnett’s multiple comparisons with the controls defined as the control group. When only two groups were available for comparison, independent means t‐tests were used. A *P*‐value of < 0.05 defined statistical significance.

### Postmortem observations

Postmortem neuropathologic diagnostic confirmation was obtained for a total of 80 individuals. Forty patients had a diagnosis of LB synucleinopathy (38 PD, 2 PAF), 5 had MSA, and 35 were controls. Postmortem neuropathologic descriptions were provided by the Banner Sun Health Research Institute (29 LB disease with neuropathologically demonstrated LBs in the brainstem, 27 controls) or the Laboratory of Pathology at the NIH Clinical Center (11 LB disease, 5 MSA, 8 controls).

Assays of norepinephrine in tissue samples were conducted by the Autonomic Medicine Section at the NIH, as described previously.^24^ The assay personnel were blinded as to the individual diagnosis until the data were tabulated. Norepinephrine concentrations were expressed as fmol/mg wet weight. Mean norepinephrine concentrations were compared between the LB and non‐LB groups by independent‐means t‐tests. Postmortem data were not available for the stomach in any of the groups because of rapid postmortem autolysis. A *P*‐value < 0.05 defined statistical significance.

## Results

### ^18^F‐DA–derived radioactivity

**Figure 1:**
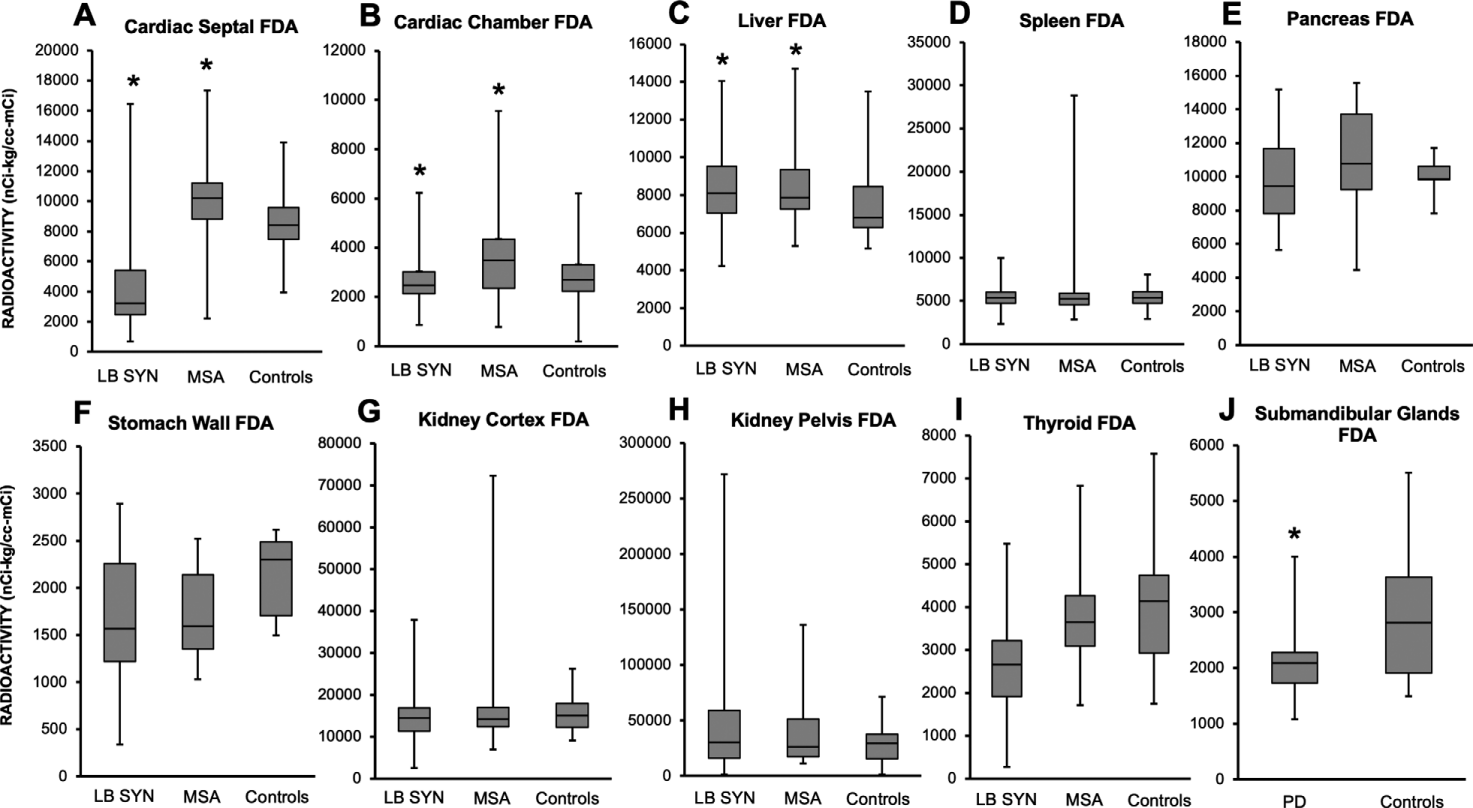
^18^F‐Dopamine (^18^F‐DA‐)–derived radioactivity in the cardiac septum (panel A), left ventricular (LV) chamber (panel B), liver (panel C), spleen (panel D), pancreas (panel E), stomach (panel F), kidney cortex (panel G), kidney pelvis (panel H), thyroid (panel I), and submandibular glands (panel J) in the patient groups and controls. In the box plots, the boundary of the box closest to zero indicates the 25th percentile, a black line within the box marks the median, and the boundary of the box farthest from zero indicates the 75th percentile. Whiskers above and below the box indicate the minimum and maximum values. Patients with LB synucleinopathies (LB SYN) had decreased septal^18^F‐DA–derived radioactivity compared to controls (*P* < 0.0001).Cardiac septal^18^F‐DA–derived radioactivity was increased in multiple system atrophy (MSA) compared to controls (*P* = 0.02).^18^F‐DA–derived radioactivity in the LV chamber was higher in MSA than in controls (*P* = 0.01).^18^F‐DA–derived radioactivity in the liver was increased in LB SYN (*P* = 0.01) and MSA (*P* = 0.03) compared to controls, whereas submandibular gland^18^F‐DA–derived radioactivity was decreased in Parkinson’s disease (PD) compared to controls (*P* = 0.04)

Patients with LB synucleinopathies had decreased ^18^F‐DA–derived radioactivity in the cardiac septum compared to controls (*P* < 0.0001, Figure 1). Septal ^18^F‐DA–derived radioactivity was increased in MSA patients compared to controls (*P* = 0.02, Figure 1). Patients with MSA also had higher ^18^F‐DA–derived radioactivity in the LV chamber (*P* = 0.01, Figure 1). Numerical data for ^18^F‐DA–derived radioactivity concentrations in body organs in synucleinopathies are summarized in a supplementary table (Supplementary data).

^18^F‐DA–derived radioactivity in the liver was higher in LB synucleinopathies (*P* = 0.01) and MSA (*P* = 0.03) than in controls (Figure 1). The groups did not differ in radioactivity in the spleen, pancreas, stomach, renal cortex, renal pelvis, or thyroid. ^18^F‐DA–derived radioactivity was decreased in the submandibular glands in LB synucleinopathies compared to the controls (*P* = 0.04, Figure 1).

### Postmortem data

**Figure 2:**
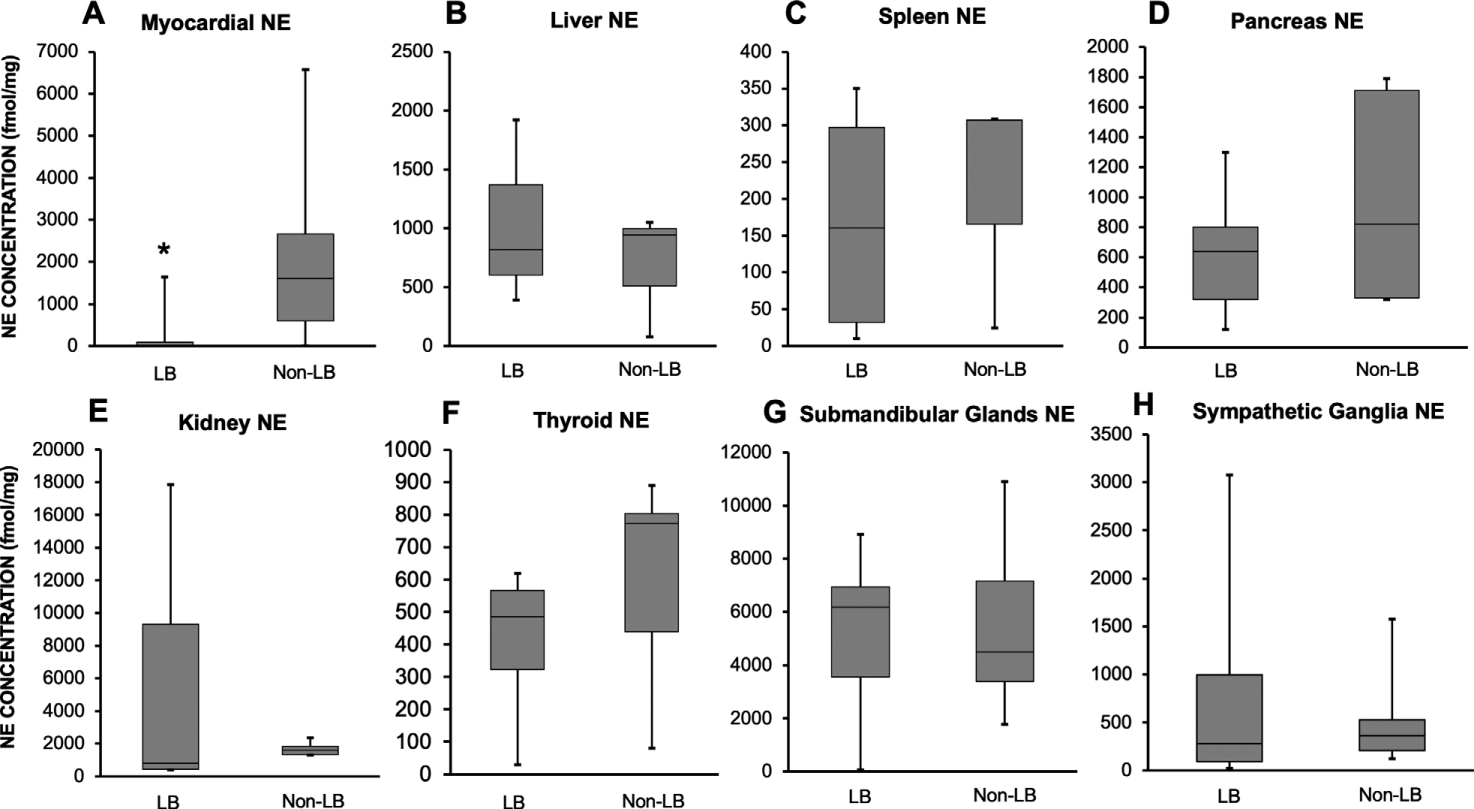
Postmortem tissue norepinephrine (NE) concentrations in apical left ventricular myocardium (panel A), liver (panel B), pancreas (panel C), spleen (panel D), kidney (panel E) thyroid (panel F), submandibular glands (panel G), and sympathetic ganglion (panel H) in the Lewy body (LB) and non‐LB groups. In the box plots, the boundary of the box closest to zero indicates the 25th percentile, a black line within the box marks the median, and the boundary of the box farthest from zero indicates the 75th percentile. Whiskers above and below the box indicate the minimum and maximum values. Myocardial NE was significantly lower in the LB group compared to the non‐LB group (*P* < 0.0001). There was no difference in tissue NE between the two groups for the liver, spleen, pancreas, thyroid, submandibular glands, and sympathetic ganglia

Myocardial norepinephrine was substantially decreased in the LB group (P < 0.0001; Figure 2) compared to the non‐LB (MSA and controls) group. The groups did not differ in mean norepinephrine concentrations in the liver, spleen, pancreas, thyroid, submandibular glands, or sympathetic ganglia (Figure 2). Mean ± SEM tissue norepinephrine concentrations in the different organs in the patients with LB versus non‐LB groups are summarized in a supplementary table (supplementary data).

### Validation of ^18^F‐DA PET scanning as a biomarker of myocardial noradrenergic innervation

**Figure 3:**
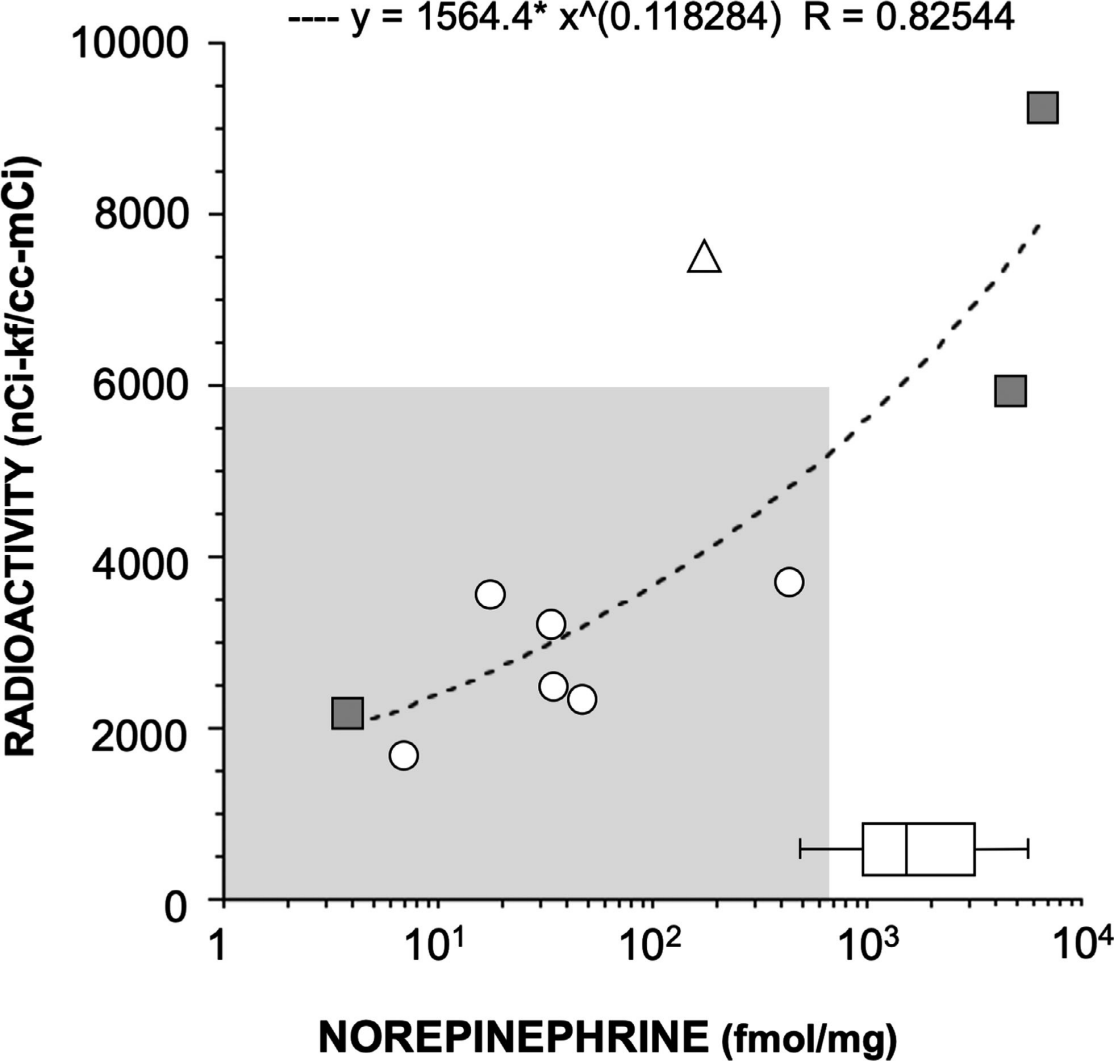
Scatter plot relating individual values for in vivo cardiac septal^18^F‐dopamine(^18^F‐DA)–derived radioactivity and postmortem myocardial norepinephrine (NE). Six subjects had a diagnosis of LB synucleinopathy (circle), three MSA (gray filled squares), and one subject with chronic neurogenic OH (triangle) (r = 0.8). The horizontal box plot represents postmortem myocardial NE concentration in all available controls. The boundary of the box plot closest to zero indicates the 25^th^percentile, a black line within the box marks the median, and the boundary of the box farthest from zero indicates the 75^th^percentile. All patients with LB synucleinopathies had low postmortem myocardial NE concentration and low septal^18^F‐DA–derived radioactivity (below 6000 nCi‐kg/cc‐mCi). One patient with MSA (empty square) had low postmortem myocardial NE concentration and low septal^18^F‐DA–derived radioactivity

A total of 11 patients (3 PD + OH, 3 PAF, 4 MSA, 1 with persistent neurogenic orthostatic hypotension without evidence of sympathetic noradrenergic deficiency) had both cardiac ^18^F‐DA PET scanning and postmortem data. Myocardial tissue was available from assayed 10 patients (Table 1). There was a strong positive correlation between ^18^F‐DA–derived radioactivity and myocardial tissue norepinephrine content (r = 0.8, Figure 3). All the patients with a diagnosis of LB synucleinopathy had both low cardiac ^18^F‐DA–derived radioactivity and low tissue norepinephrine compared to controls. One patient with pathologically confirmed MSA had low myocardial tissue norepinephrine and low ^18^F‐DA–derived radioactivity. The patient with neurogenic orthostatic hypotension and no neuroimaging evidence of sympathetic noradrenergic deficiency had low myocardial norepinephrine content and no LB pathology.

**Table 1:** Demographics and pathology of subjects with both in vivo neuroimaging and postmortem data

Subject	Clinical diagnosis	Sex	Age at onset *y*	Disease duration *y*	Septum FDA *nCi‐kg/cc‐mCi*	Myocardium NE *fmol/mg*	Pathology				
							PMI *h*	LBs	GCIs	AD	Other
1	PD + OH	F	69	14.6	2293	36	20	SN+	No	Hirano bodies: H+	No
2	PD + OH	F	61	22.5	1765	48	30	Intraneuronal α‐synuclein in brainstem and sympathetic ganglia			
3	PD + OH	M	75	2.8	3729	431	24	SN ++	No	No	No
4	PAF	F	48	35.6	1733	7	<24h	SN+ LC+++	No	No	Marked cerebral atherosclerosis
5	PAF	M	66	3.1	3101	18	23	Am+ CG+ SN++ Brainstem++	No	NFT: H+, EC+ BA: no	No
6	PAF	M	53	15.9	3218	34	12	SN+	No	NP: FC+, TC+, PC+ No NFT	No
7	NOH	M	50	7.7	7434	184	15	No	No	No	No
8	MSA‐C	M	46	11	8913	NA	<24h	No	Midbrain+++ Pons+++ Cereb.+++ SN+	No	No
9	MSA‐P	M	60	6.2	11308	6569	8	No	SN+++ St.+++ NB+	Tau: Th+, NB+, Am+ BA: Am+, Th+, BG+	No
10	MSA‐P	M	69	3.2	2219	4	8	No	SN+, CG+, H+, EC+	No	No SYN in heart, SG
11	MSA‐P	M	71	6.7	5924	4428	<24h	Final Pathology report requested – unavailable at the time of submission			

AD, Alzheimer’s disease pathology; Am, amygdala; BA, beta‐amyloid; Cereb., cerebellum; CG, cingular cortex; EC, entorhinal cortex; F, female; FC, frontal cortex; GCIs, glial cytoplasmic inclusions; H, hippocampus; LBs, Lewy bodies; M, male; M; MSA‐C, multiple system atrophy subtype cerebellar; MSA‐P, multiple system atrophy subtype parkinsonian; NB, nucleus basalis; NFT, neurofibrillary tangles; NP, neuritic plaques; NOH, neurogenic orthostatic hypotension; PAF, pure autonomic failure; PC, parietal cortex; PD + OH, Parkinson’s disease with orthostatic hypotension; PMI, postmortem interval; SG, sympathetic ganglia; St, striatum; SN, substantia nigra; TC, temporal cortex; Th, thalamus; SYN, synuclein immunoreactivity. +, mild or focal pathological burden; ++, moderate pathological burden; +++, severe pathological burden.

## Discussion

The results of this study show that LB forms of alpha‐synucleinopathy involve peripheral noradrenergic deficiency that is especially prominent in the heart, by both in vivo ^18^F‐DA PET scanning and postmortem neurochemistry. A major and unique strength of the study was the assays of tissue norepinephrine contents in tissues from a variety of extracranial organs.

### Validation of ^18^F‐DA PET scanning as a biomarker of cardiac noradrenergic innervation

We validated low ^18^F‐DA–derived radioactivity as a biomarker of the cardiac noradrenergic deficiency by comparing in vivo radioactivity with postmortem myocardial norepinephrine content in the same patients. Analogously, heart/mediastinum ratios of ^123^I‐metaiodobenzylguanidine (^123^I‐MIBG)–derived radioactivity have been found to correlate positively with myocardial immunoreactive tyrosine hydroxylase.^25^

It should be noted that low ^18^F‐DA–derived radioactivity does not imply sympathetic noradrenergic denervation. A functional abnormality in intact sympathetic nerves could produce the same abnormality.^26^, ^27^ For instance, a recent multi‐tracer imaging study found that PAF patients have a combination of a moderate amount of loss of cardiac noradrenergic innervation and a substantial vesicular storage defect in residual neurons.^28^

### Profound cardiac norepinephrine deficiency in LB synucleinopathies

Interventricular septal myocardial ^18^F‐DA–derived radioactivity was decreased in three forms of LB synucleinopathy – PD + OH, PD No OH, and PAF. These results fit with those from numerous previous reports based on cardiac sympathetic neuroimaging by ^18^F‐DA or ^123^I‐MIBG scanning in these conditions.^23^, ^29^ The current data provide postmortem confirmation of drastic myocardial noradrenergic deficiency in LB disease. Mean myocardial norepinephrine content was decreased by 97% from that in controls.

In contrast, septal ^18^F‐DA–derived radioactivity was significantly increased in the group with the non‐LB synucleinopathy MSA. The increased radioactivity might reflect decreased postganglionic sympathetic nerve traffic, which tends to increase tissue contents of the tracer.^27^

Mean myocardial norepinephrine content was normal in a small group of MSA patients.

No previous study has reported on myocardial norepinephrine content in MSA, possibly because MSA is a rare disease, and autopsy studies rarely involve extracranial tissue harvesting. A patient with pathologically confirmed MSA had both PET and postmortem neurochemical data demonstrating severe cardiac noradrenergic deficiency. This patient had an increased “synuclein index” in arrector pili muscles during life, indicating a peripheral intraneuronal synucleinopathic process.^30^ We speculate that the patient had an early peripheral LB process. Unfortunately, in this patient postmortem harvesting of sympathetic ganglion tissue was not done. Overall, the results are consistent with other studies where most MSA patients have biomarkers of intact cardiac sympathetic innervation, although rare cases of MSA with neuroimaging evidence of cardiac noradrenergic deficiency have been reported.^31^, ^32^, ^33^

### No evidence for decreased norepinephrine contents in extracardiac organs in LB synucleinopathies

In this study, tissue norepinephrine contents in extracardiac organs were similar in the LB and non‐LB groups. Alpha‐synuclein deposition in salivary glands has been proposed as a potential biomarker of early autonomic involvement in PD.^34^, ^35^, ^36^ Despite neuroimaging evidence that suggests noradrenergic deficiency in the submandibular gland in PD, in this study tissue norepinephrine content was normal in the LB synucleinopathy group. Discordance between neuroimaging and tissue norepinephrine data might reflect a functional abnormality that decreases neuronal uptake of sympathetic imaging agents without actual denervation. No previous studies have assessed submandibular gland norepinephrine contents in synucleinopathies. A recent report noted lower ^123^I‐MIBG–derived radioactivity in the parotid and submandibular glands in PD than in control subjects.^37^ The authors concluded that decreased ^123^I‐MIBG uptake could be explained by alpha‐synuclein pathology in the superior cervical ganglion.^37^ This study did not include postmortem neuropathological or neurochemical data.

Our group and others reported previously that patients with PD and OH have decreased ^18^F‐DA–derived radioactivity in the thyroid.^13^, ^38^, ^39^ Taki et al. did not find evidence of reduced ^123^I‐MIBG–derived radioactivity in PD patients without autonomic dysfunction.^40^ The different results could be due to sample size and the fact that only PD patients without autonomic failure were included.^40^ In the current study, postmortem thyroid norepinephrine concentrations did not differ between the LB and non‐LB groups. No previous reports have described thyroid norepinephrine contents in synucleinopathies.

The role of the sympathetic nervous system in the regulation of pancreatic functions is incompletely understood. Splanchnic nerve stimulation has been shown to decrease plasma insulin levels, possibly via direct actions of norepinephrine on pancreatic beta‐cells.^41^ A link between disrupted brain insulin signaling and PD has been suggested by an increased rate of subsequent PD following type 2 diabetes mellitus in a large cohort study.^42^ Furthermore, cytoplasmic phosphorylated alpha‐synuclein has been found in the pancreas of subjects with synucleinopathies or with diabetes melitus,^43^ and there is evidence for alpha‐synuclein inhibiting insulin secretion and dopamine synthesis.^44^ Our study is the first to report on in vivo imaging of noradrenergic innervation and postmortem tissue norepinephrine in the human pancreas. By both methodologies the current findings do not suggest pancreatic noradrenergic deficiency in synucleinopathies. Further studies should further characterize the role of α‐synuclein on pancreatic neurotransmitter, hormonal, and autocrine–paracrine functions.

### Larger decrease in norepinephrine in myocardium than in sympathetic ganglion tissue in LB synucleinopathies

The large decrease in myocardial tissue norepinephrine in LB compared to non‐LB controls was not accompanied by similarly decreased tissue norepinephrine in sympathetic ganglion tissue. There are a variety of explanations for this difference. One for which there is relevant literature is that the pathogenetic process may proceed in a centripetal fashion.^45^ It has been suggested that synucleinopathies may involve decreased axonal transport relatively early in the disease process,^46^, ^47^ and since vesicles and vesicle‐associated proteins are delivered by axonal transport, there could be decreased populations of functionally intact storage vesicles at terminals in the target tissue.^48^, ^49^

### Why are cardiac sympathetic nerves susceptible in LB diseases?

The heart relies almost exclusively on aerobic oxidation for the generation of energy. Therefore, in comparison to other organs, sympathetic nerves in the heart might be particularly sensitive to alterations of mitochondrial functions and oxidative stress; both have been shown to play a role in the pathogenesis of PD.^50^ The heart also stands out in the magnitude of oxidative deamination of cytoplasmic catecholamines. Thus, between the arterial and coronary sinus, the plasma concentration of 3,4‐dihydroxyphenylglycol, the main product of oxidative deamination of norepinephrine in sympathetic nerves, approximately doubles.^51^, ^52^ Moreover, unlike other organs, about 80% of norepinephrine in coronary arterial plasma is removed in the passage through the heart.^52^ If there were a circulating sympathetic neurotoxin there might be greater damage to sympathetic nerves in the heart than in other organs.

### Limitations

Except for 10 patients, the subject cohort that underwent in vivo neuroimaging was different from the cohort that underwent postmortem tissue neurochemistry. Therefore, there is a risk of imperfect concordance across subjects for the organs sampled. Comprehensive clinical data (e.g., clinical diagnosis, disease duration) or other pathologic findings (e.g., LB subtype, NIA‐AA Alzheimer type pathology, DLB criteria, or co‐morbid TDP‐43 or cerebrovascular pathologies) were unavailable for most of the cohort that underwent postmortem tissue harvesting.

We did not obtain gastrointestinal tissue samples for neurochemical assays because of rapid postmortem autolysis, which would obviate obtaining valid data about tissue NE contents. Alpha‐synuclein deposition and LBs have been reported in the gastrointestinal tract,^53^ but whether PD patients have increased gastrointestinal alpha‐synuclein deposition has been questioned. No differences between PD and controls have been reported in numbers of tyrosine hydroxylase‐positive myenteric and submucosal neurons in gastrointestinal organs.^54^, ^55^ One should bear in mind that the occurrence of LB pathology does not imply catecholamine deficiency. The status of noradrenergic innervation in the enteric nervous system in LB diseases therefore remains unknown. This is an important matter for future research involving gastrointestinal tissue sampling with short postmortem intervals.

We did not assay alpha‐synuclein and norepinephrine in the same tissue samples. The focus of the present study was on noradrenergic innervation, not on the putative pathophysiologic role of intraneuronal alpha‐synuclein deposition. This is a matter of current research.

## Conclusion and outlook

In conclusion, LB synucleinopathies entail cardioselective peripheral noradrenergic deficiency. It is possible that a better understanding of bases for this selectivity may apply also to the unusual susceptibility of nigrostriatal dopaminergic neurons in PD.

## Authors’ Contributions

All authors made substantial contributions. Guillaume Lamotte contributed to the conception and design of the study, acquisition, analysis and interpretation of data, drafting the manuscript and preparing the figures, and reviewing the manuscript; Patricia Sullivan and Courtney Holmes contributed to the analysis and interpretation of data and reviewing the manuscript; Abhishek Lenka contributed to the interpretation of data, reviewing the manuscript; David S. Goldstein contributed to the conception and design of the study, acquisition, analysis and interpretation of data, preparing the figures, and reviewing the manuscript.

## Conflicts of Interest

The Corresponding Author affirms that none of the authors has a conflict of interest.

## Supporting information


**Table S1.** Reports numerical data for ^18^F‐Dopamine (^18^F‐DA‐)–derived radioactivity in body organs in synucleinopathies and controls. Cardiac septal ^18^F‐DA–derived radioactivity was decreased in Lewy body synucleinopathies (Parkinson’s disease with orthostatic hypotension (PD + OH), PD without OH (OH), and pure autonomic failure (PAF)) compared to controls. Cardiac septal and left ventricular chamber ^18^F‐DA–derived radioactivity concentrations were increased in multiple system atrophy (MSA) compared to controls. Liver ^18^F‐DA–derived radioactivity was increased in PD + OH, PAF, and MSA compared to controls. ^18^F‐DA–derived radioactivity concentration in submandibular glands was decreased in PD compared to controls. The groups did not differ in radioactivity in the spleen, pancreas, stomach, renal cortex, renal pelvis, or thyroid.
**Table S2.** Reports numerical data for postmortem tissue norepinephrine (NE) in the different organs of interest in the Lewy body (LB) group (Parkinson’s disease = 37, pure autonomic failure = 3) and the non‐Lewy body group (controls = 35, multiple system atrophy = 5). Myocardial NE was substantially decreased in the LB group (*P* < 0.0001) compared to the non‐LB (MSA and controls) group.
