# Supplementary material for: Cardioselective peripheral noradrenergic deficiency in Lewy body synucleinopathies
Source: Ann Clin Transl Neurol. 2020 Nov 20;7(12):2450–60. doi: 10.1002/acn3.51243 (PMC7732242; doi:10.1002/acn3.51243)
Supplement: Supplementary file 1 — Table S1. Reports numerical data for 18F‐Dopamine (18F‐DA‐)–derived radioactivity in body organs in synucleinopathies and controls. Cardiac septal 18F‐DA–derived radioactivity was decreased in Lewy body synucleinopathies (Parkinson’s disease with orthostatic hypotension (PD + OH), PD without OH (OH), and pure autonomic failure (PAF)) compared to controls. Cardiac septal and left ventricular chamber 18F‐DA–derived radioactivity concentrations were increased in multiple system atrophy (MSA) compared to controls. Liver 18F‐DA–derived radioactivity was increased in PD + OH, PAF, and MSA compared to controls. 18F‐DA–derived radioactivity concentration in submandibular glands was decreased in PD compared to controls. The groups did not differ in radioactivity in the spleen, pancreas, stomach, renal cortex, renal pelvis, or thyroid. Table S2. Reports numerical data for postmortem tissue norepinephrine (NE) in the different organs of interest in the Lewy body (LB) group (Parkinson’s disease = 37, pure autonomic failure = 3) and the non‐Lewy body group (controls = 35, multiple system atrophy = 5). Myocardial NE was substantially decreased in the LB group (P < 0.0001) compared to the non‐LB (MSA and controls) group. [file ACN3-7-2450-s001.docx]

**SUPPLEMENTARY TABLES**

**Table 1. 18F-Dopamine- (18F-DA-)-derived radioactivity in body organs in synucleinopathies.**

| **18F-DA derived radioactivity (nCi-kg/cc-mCi), mean (± SEM)** | | | | | |
| --- | --- | --- | --- | --- | --- |
|  | **PD+OH** | **PD No OH** | **PAF** | **MSA** | **Controls** |
| Cardiac Septum | 3053 (218)* | 5733 (443)* | 3313 (196)* | 10164 (247)* | 9093 (306) |
| LV Chamber | 2457 (114) | 2658 (110) | 2701 (138) | 3537 (172)* | 3014 (157) |
| Liver | 8577 (232)* | 7907 (217) | 8881 (358)* | 8366 (177)* | 7621 (238) |
| Spleen | 5259 (154) | 5337 (154) | 5702 (197) | 5536 (308) | 5405 (149) |
| Pancreas | 10635 (886) | 9027 (780) | 10153 (709) | 11057 (1001) | 9993 (404) |
| Stomach | 1603 (402) | 1614 (227) | 1600 (159) | 1735 (158) | 2116 (163) |
| Kidney Cortex | 13849 (948) | 13608 (615) | 15544 (820) | 15692 (1214) | 16442 (822) |
| Kidney Pelvis | 33538 (4199) | 50928 (8837) | 57346 (10381) | 38521 (3926) | 44484 (7734) |
| Thyroid | 2404 (228) | 2971 (175) | 2924 (223) | 3750 (204) | 4114 (670) |
| Submandibular glands | 2091 (0)* | 2022 (179)* | N/A | N/A | 2939 (371) |

Abbreviations: LV = left ventricular, PD+OH = Parkinson’s disease with neurogenic orthostatic hypotension, PD No OH = Parkinson’s disease without orthostatic hypotension, PAF = pure autonomic failure, MSA = multiple system atrophy. * p < 0.05, differences between groups were assessed by Dunnett's multiple comparisons with the controls defined as the control group.

**Table 2. Post-mortem tissue norepinephrine (NE) in the different organs of interest in the Lewy body group (PD = 37, PAF = 3) and the non-Lewy body group (controls = 35, MSA = 5)**

|  | **Post-mortem tissue NE (fmol/mg wet weight), mean (± SEM), N** | |
| --- | --- | --- |
|  | **Lewy body diseases** | **Non Lewy body diseases/controls** |
| Myocardium | 157 (56), N = 40* | 1927 (246), N = 40 |
| Liver | 1043 (372), N = 3 | 690 (250), N = 3 |
| Spleen | 170 (74), N = 4 | 213 (77), N = 3 |
| Pancreas | 614 (140), N = 7 | 994 (288), N = 5 |
| Kidney | 6243 (4741), N = 3 | 1564 (293), N = 4 |
| Thyroid | 405 (114), N = 4 | 597 (134), N = 5 |
| Submandibular glands | 5361 (614), N = 18 | 5139 (599), N = 17 |
| Sympathetic ganglia | 657 (324), N = 13 | 575 (172), N = 14 |

Abbreviations: PAF = Pure autonomic failure, PD = Parkinson’s disease, MSA = Multiple system atrophy. * p < 0.05, differences between the two groups were assessed by independent t-test
